# Prop-2-enyl 6-amino-5-cyano-4-(4-iso­propylphen­yl)-2-methyl-4*H*-pyran-3-carboxyl­ate

**DOI:** 10.1107/S2414314626001707

**Published:** 2026-02-24

**Authors:** C. Udhaya Kumar, G. Anantha Krishnan, P. Jamuna Rani, M. Velayutham Pillai, T. Mohandas

**Affiliations:** a Department of Chemistry, Mahendra Engineering College, Mahendhirapuri, Namakkal, Tamilnadu-637503, India; b Department of Physics, Saranathan College of Engineering, Panjappur, Tiruchirappalli, Tamilnadu-620012, India; c Department of Chemistry, Mahendra Institute of Technology (Autonomous), Mallasamudram, Namakkal, Tamilnadu-637503, India; d Post Graduate Department of Chemistry, Nallamuthu Gounder Mahalingam College, Pollachi, Tamil Nadu-642001, India; e Department of Physics, J. J. College of Engineering and Technology, Tiruchirappalli, Tamilnadu-620009, India; Howard University, USA

**Keywords:** crystal structure, pyran, hydrogen bonding

## Abstract

In the title compound, C_20_H_22_N_2_O_3_, the 4*H-*pyran ring adopts a boat conformation. The dihedral angle between the phenyl and pyran rings is 87.8 (18)°. In the crystal, mol­ecules are linked by N—H⋯O and N—H⋯N hydrogen bonds. The allyl side chain is disordered in a 0.582 (13):0.418 (13) ratio.

## Abstract 2

In the title compound, the 4*H-*pyran ring adopts a boat conformation. The dihedral angle between the phenyl and pyran rings is 87.8 (18)°. In the crystal, mol­ecules are linked by N—H⋯O and N—-H⋯N hydrogen bonds.

## Graphical abstract

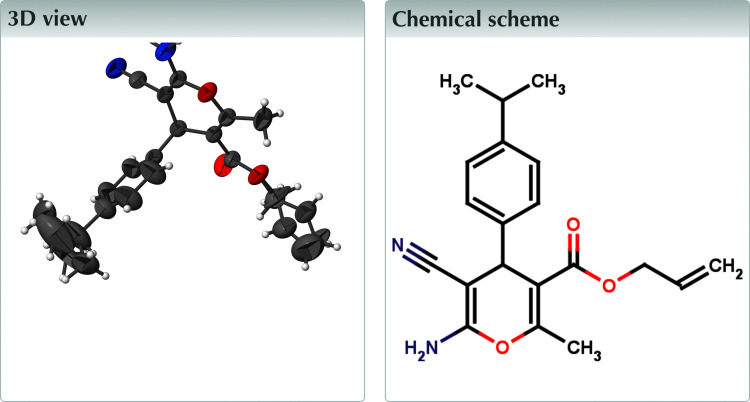

## Structure description

2-Amino-4*H*-pyran derivatives are an important class of heterocycles, which are of considerable inter­est due to their useful biological properties including anti­microbial (Saga Kitamura *et al.*, 2006 ▸), anti­fungal (Tangmouo *et al.*, 2006 ▸), cancer therapy (Cocco *et al.*, 2003 ▸) and central nervous system activity (Eiden *et al.*, 1991 ▸). Pyran derivatives constitute a useful class of heterocyclic compounds, which are widely distributed in nature (Moriguchi *et al.*, 1997 ▸). Some 2-amino-4*H*-pyrans are used as photoactive materials (Armesto *et al.*, 1989 ▸), pigments (Rideout *et al.*, 1976 ▸) and potentially biodegradable agrochemicals (Kumar *et al.*, 2009 ▸). Pyran­ochalcones have been reported to exhibit anti-mutagenic, anti­microbial, anti­ulcer and anti-tumor activities (Lee *et al.*, 2007 ▸). Polyfunctionalized 4*H*-pyran, a major constituent of many natural products (Hatakeyama *et al.*, 1988 ▸; Singh *et al.*, 1996 ▸; Martín *et al.*, 1993 ▸) is known for its wide array of biological activities. Recent findings have suggested that the compounds having a 4*H*-pyran core are useful for the treatment of Alzheimer’s, schizophrenia and myoclonus diseases.

**Figure 1:**
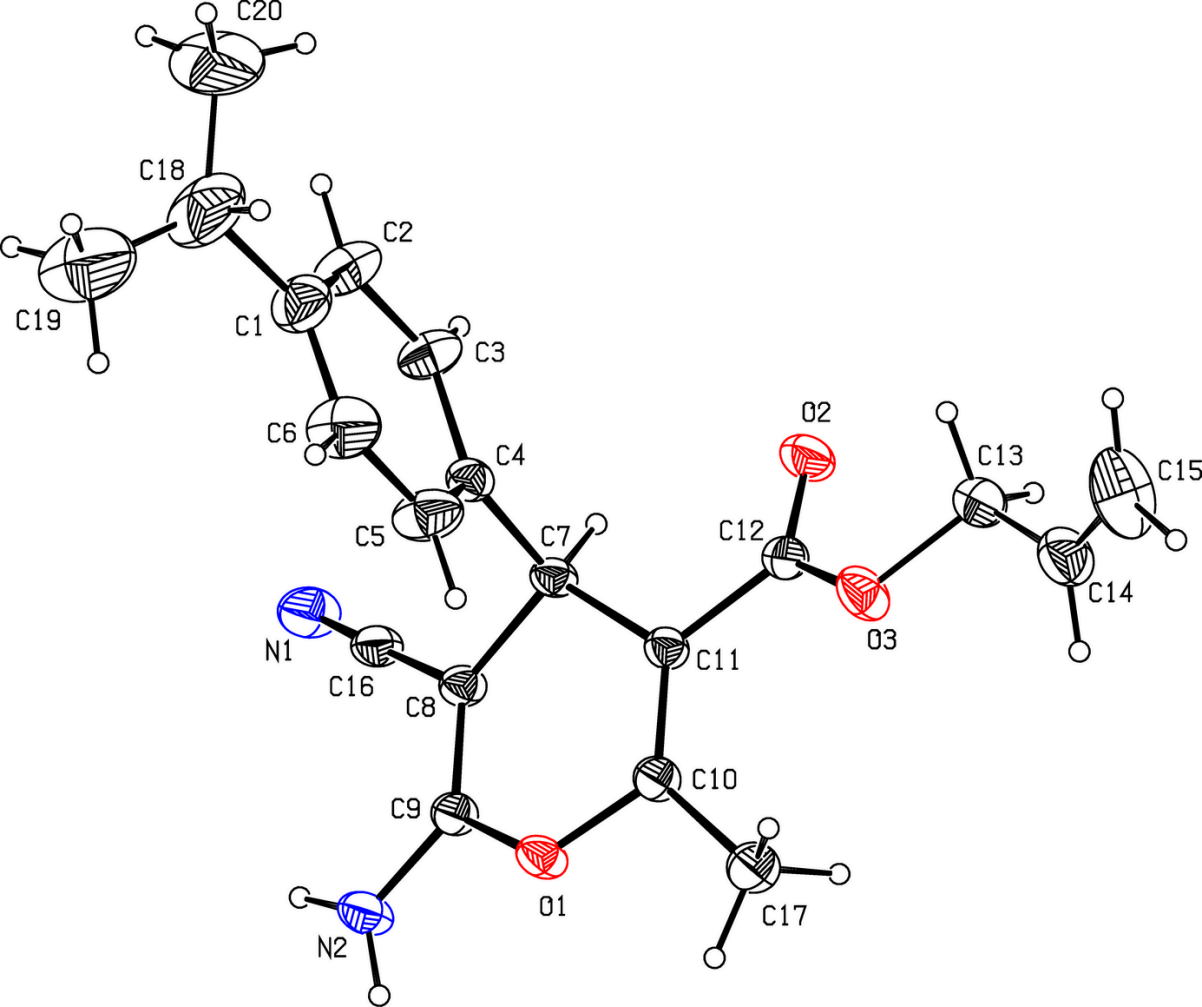
The mol­ecular structure of the title compound with displacement ellipsoids drawn at the 20% probability level.

The 4*H-*pyran ring in the title compound (Fig. 1 ▸) exhibits a boat conformation with puckering parameters *Q* = 0.252 (3) Å, θ = 79.2 (7)° and φ = 168.6 (7)° (Cremer & Pople, 1975 ▸). In the 4*H*-pyran ring, atoms O1 and C7 have the maximum deviations of 0.127 (2) and 0.152 (3)Å, respectively, from the mean plan. The dihedral angle between phenyl to pyran ring is found to be 87.80 (18)°. The allyl side chain is disordered with a site occupancy ratio of 0.582 (13):0.418 (13). Atoms C19 and C20 are also disordered [occupancy ratio of 0.503 (12):0.497 (12)]. The torsion angles C7—C11—C12—O3, C5—C4—C7—C8 and C18—C1—2—C3 are −156.9 (2), −78.7 (4) and 177.7 (5)° respectively.

**Figure 2:**
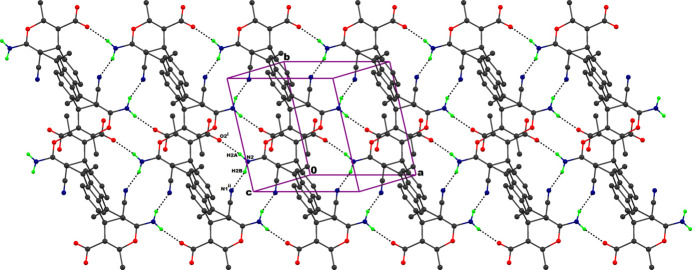
The packing diagram of the title compound showing the N—H⋯N and N—H⋯O inter­actions.

In the crystal, mol­ecules are linked *via* N2—H2*A*⋯O2 and N2—H2*B*⋯N1 hydrogen bonds, resulting in centrosymmetric dimers with adjacent 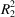
(12) and 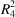
(20) ring motifs running parallel to the *a* axis (Table 1 ▸, Fig. 2 ▸). For a related structure, see: Mohendas *et al.* (2015 ▸).

## Synthesis and crystallization

A mixture of 4-iso­propyl­benzaldehyde (1.0 mmol), malono­nitrile (1.0 mmol), allyl 3-oxo­butano­ate (1.0 mmol), and a few drops of piperidine was stirred magnetically in 30 ml of absolute ethanol at 80°C for the required period of time (90 min). The progress of the reaction was monitored by TLC. After completion of the reaction, the reaction mixture was allowed to cool to room temperature and the solvent was evaporated. The solid thus obtained was collected and washed with cold water and recrystallized from ethanol solution to get the pure product (yield 82%).

## Refinement

Crystal data, data collection and structure refinement details are summarized in Table 2 ▸.

## Supplementary Material

Crystal structure: contains datablock(s) global, I. DOI: 10.1107/S2414314626001707/bv4058sup1.cif

Structure factors: contains datablock(s) I. DOI: 10.1107/S2414314626001707/bv4058Isup2.hkl

Supporting information file. DOI: 10.1107/S2414314626001707/bv4058Isup3.cml

CCDC reference: 1916641

Additional supporting information:  crystallographic information; 3D view; checkCIF report

Additional supporting information:  crystallographic information; 3D view; checkCIF report

## Figures and Tables

**Table 1:** Hydrogen-bond geometry (Å, °)

*D*—H⋯*A*	*D*—H	H⋯*A*	*D*⋯*A*	*D*—H⋯*A*
N2—H2*A*⋯O2^i^	0.87 (4)	2.04 (4)	2.896 (4)	170 (3)
N2—H2*B*⋯N1^ii^	0.98 (4)	2.08 (4)	3.034 (4)	163 (3)

Symmetry codes: (i) 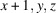; (ii) 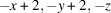.

**Table 2:** Experimental details

Crystal data	
Chemical formula	C_20_H_22_N_2_O_3_
*M*_r_	338.32
Crystal system, space group	Triclinic, *P* 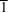
Temperature (K)	293
*a*, *b*, *c* (Å)	8.2556 (5), 9.2288 (5), 13.5978 (8)
α, β, γ (°)	102.246 (3), 102.970 (3), 103.989 (3)
*V* (Å^3^)	939.96 (10)
*Z*	2
Radiation type	Mo *K*α
μ (mm^−1^)	0.08
Crystal size (mm)	0.20 × 0.20 × 0.15

Data collection	
Diffractometer	Bruker Kappa APEXII CCD
Absorption correction	Multi-scan (*SADABS*; Krause *et al.*, 2015 ▸)
*T*_min_, *T*_max_	0.984, 0.984
No. of measured, independent and observed [*I* > 2σ(*I*)] reflections	17196, 3305, 2093
*R*_int_	0.031
(sin θ/λ)_max_ (Å^−1^)	0.595

Refinement	
*R*[*F*^2^ > 2σ(*F*^2^)], *wR*(*F*^2^), *S*	0.067, 0.252, 1.02
No. of reflections	3305
No. of parameters	281
No. of restraints	98
Δρ_max_, Δρ_min_ (e Å^−3^)	0.41, −0.23

Computer programs: *APEX2*, *SAINT* and *XPREP* (Bruker, 2008 ▸), *SHELXS97* (Sheldrick, 2008 ▸), *ORTEP-3 for Windows* (Farrugia, 2012 ▸), *SHELXL97* (Sheldrick, 2008 ▸) and *PLATON* (Spek, 2020 ▸).

## full crystallographic data

### Prop-2-enyl 6-amino-5-cyano-4-(4-isopropylphenyl)-2-methyl-4H-pyran-3-carboxylate Crystal data

**Table:**

C_20_H_22_N_2_O_3_	*Z* = 2
*M**_r_* = 338.32	*F*(000) = 360
Triclinic, *P*1	*D*_x_ = 1.195 Mg m^−^^3^
Hall symbol: -P 1	Mo *K*α radiation, λ = 0.71073 Å
*a* = 8.2556 (5) Å	Cell parameters from 3305 reflections
*b* = 9.2288 (5) Å	θ = 2.4–25.0°
*c* = 13.5978 (8) Å	µ = 0.08 mm^−^^1^
α = 102.246 (3)°	*T* = 293 K
β = 102.970 (3)°	Block, orange
γ = 103.989 (3)°	0.20 × 0.20 × 0.15 mm
*V* = 939.96 (10) Å^3^	

### Prop-2-enyl 6-amino-5-cyano-4-(4-isopropylphenyl)-2-methyl-4H-pyran-3-carboxylate Data collection

**Table:**

Bruker Kappa APEXII CCD diffractometer	3305 independent reflections
Radiation source: fine-focus sealed tube	2093 reflections with *I* > 2σ(*I*)
Graphite monochromator	*R*_int_ = 0.031
ω and φ scan	θ_max_ = 25.0°, θ_min_ = 2.4°
Absorption correction: multi-scan (SADABS; Krause *et al.*, 2015)	*h* = −9→9
*T*_min_ = 0.984, *T*_max_ = 0.984	*k* = −10→10
17196 measured reflections	*l* = −16→16

### Prop-2-enyl 6-amino-5-cyano-4-(4-isopropylphenyl)-2-methyl-4H-pyran-3-carboxylate Refinement

**Table:**

Refinement on *F*^2^	Primary atom site location: structure-invariant direct methods
Least-squares matrix: full	Secondary atom site location: difference Fourier map
*R*[*F*^2^ > 2σ(*F*^2^)] = 0.067	Hydrogen site location: inferred from neighbouring sites
*wR*(*F*^2^) = 0.252	H-atom parameters not defined?
*S* = 1.02	*w* = 1/[σ^2^(*F*_o_^2^) + (0.1525*P*)^2^ + 0.3295*P*] where *P* = (*F*_o_^2^ + 2*F*_c_^2^)/3
3305 reflections	(Δ/σ)_max_ < 0.001
281 parameters	Δρ_max_ = 0.41 e Å^−^^3^
98 restraints	Δρ_min_ = −0.23 e Å^−^^3^

### Prop-2-enyl 6-amino-5-cyano-4-(4-isopropylphenyl)-2-methyl-4H-pyran-3-carboxylate Special details

**Table:**

Geometry. Bond distances, angles etc. have been calculated using the rounded fractional coordinates. All esds are estimated from the variances of the (full) variance-covariance matrix. The cell esds are taken into account in the estimation of distances, angles and torsion angles
Refinement. Refinement of F^2^ against ALL reflections. The weighted R-factor wR and goodness of fit S are based on F^2^, conventional R-factors R are based on F, with F set to zero for negative F^2^. The threshold expression of F^2^ > 2sigma(F^2^) is used only for calculating R-factors(gt) etc. and is not relevant to the choice of reflections for refinement. R-factors based on F^2^ are statistically about twice as large as those based on F, and R-factors based on ALL data will be even larger.The positions of the hydrogen atoms bound to the O and C atoms are identified from the difference electron density maps and their distances are geometrically optimized. The hydrogen atoms bound to the C atoms are treated as riding atoms, with d(C—H)=0.93 and *U*iso(H) = 1.2Ueq(C) for aromatic, d(C—H)=0.97 and *U*iso(H)=1.2Ueq(C) for methylene and d(C—H)=0.96 and *U*iso(H) =1.5*U*eq(C) for methyl groups.

### Prop-2-enyl 6-amino-5-cyano-4-(4-isopropylphenyl)-2-methyl-4H-pyran-3-carboxylate Fractional atomic coordinates and isotropic or equivalent isotropic displacement parameters (Å^2^)

**Table:**

	*x*	*y*	*z*	*U*_iso_*/*U*_eq_	Occ. (<1)
O1	0.8579 (3)	0.5858 (2)	0.14438 (17)	0.0552 (7)	
O2	0.2743 (3)	0.5623 (3)	0.1141 (2)	0.0751 (9)	
O3	0.3713 (3)	0.4014 (3)	0.19389 (19)	0.0674 (8)	
N1	0.8004 (4)	1.0111 (3)	0.0100 (3)	0.0778 (14)	
N2	1.0391 (4)	0.7482 (3)	0.0900 (3)	0.0647 (10)	
C1	0.6891 (6)	1.1023 (4)	0.4213 (3)	0.0841 (16)	
C2	0.5859 (7)	1.1007 (4)	0.3271 (4)	0.0903 (18)	
C3	0.5552 (5)	0.9856 (4)	0.2358 (3)	0.0713 (14)	
C4	0.6283 (4)	0.8674 (3)	0.2358 (2)	0.0503 (10)	
C5	0.7289 (6)	0.8654 (5)	0.3299 (3)	0.0861 (16)	
C6	0.7588 (7)	0.9829 (6)	0.4207 (3)	0.100 (2)	
C7	0.6031 (4)	0.7470 (3)	0.1336 (2)	0.0456 (9)	
C8	0.7606 (4)	0.7855 (3)	0.0931 (2)	0.0477 (9)	
C9	0.8827 (4)	0.7122 (3)	0.1071 (2)	0.0488 (10)	
C10	0.6928 (4)	0.5130 (3)	0.1494 (2)	0.0496 (9)	
C11	0.5705 (3)	0.5841 (3)	0.1446 (2)	0.0451 (9)	
C12	0.3916 (4)	0.5152 (3)	0.1482 (2)	0.0515 (10)	
C13	0.1931 (12)	0.3350 (14)	0.1967 (10)	0.069 (4)	0.582 (13)
C14	0.1803 (9)	0.2024 (11)	0.2375 (7)	0.082 (3)	0.582 (13)
C15	0.098 (2)	0.167 (2)	0.2976 (12)	0.123 (5)	0.582 (13)
C16	0.7845 (4)	0.9104 (3)	0.0480 (3)	0.0556 (11)	
C17	0.6902 (5)	0.3557 (4)	0.1615 (3)	0.0683 (13)	
C18	0.7268 (10)	1.2331 (7)	0.5200 (5)	0.143 (3)	
C19	0.898 (2)	1.307 (2)	0.5809 (15)	0.193 (8)	0.503 (12)
C20	0.5928 (17)	1.310 (2)	0.5260 (12)	0.221 (8)	0.503 (12)
C13'	0.1937 (18)	0.325 (3)	0.1925 (16)	0.107 (10)	0.418 (13)
C14'	0.2048 (18)	0.306 (2)	0.2966 (12)	0.107 (5)	0.418 (13)
C15'	0.145 (4)	0.210 (4)	0.337 (2)	0.166 (10)	0.418 (13)
C19'	0.750 (2)	1.1939 (13)	0.6162 (8)	0.139 (6)	0.497 (12)
C20'	0.896 (3)	1.3577 (17)	0.5233 (13)	0.185 (7)	0.497 (12)
H6	0.82917	0.97982	0.48359	0.1201*	
H7	0.50156	0.74924	0.08098	0.0547*	
H2	0.53469	1.17971	0.32443	0.1086*	
H2A	1.099 (4)	0.683 (4)	0.093 (2)	0.061 (9)*	
H2B	1.071 (4)	0.834 (4)	0.059 (3)	0.071 (10)*	
H3	0.48363	0.98839	0.17315	0.0858*	
H5	0.77763	0.78500	0.33313	0.1035*	
H17A	0.80146	0.34067	0.16206	0.1028*	
H17B	0.66665	0.34749	0.22647	0.1028*	
H17C	0.60086	0.27764	0.10388	0.1028*	
H18	0.69598	1.16630	0.56442	0.1721*	0.503 (12)
H19A	0.96769	1.23954	0.56711	0.2895*	0.503 (12)
H19B	0.94204	1.40151	0.56393	0.2895*	0.503 (12)
H19C	0.90299	1.32994	0.65399	0.2895*	0.503 (12)
H20A	0.48259	1.24293	0.47879	0.3324*	0.503 (12)
H20B	0.58441	1.33355	0.59654	0.3324*	0.503 (12)
H20C	0.62346	1.40512	0.50647	0.3324*	0.503 (12)
H13A	0.11358	0.30371	0.12621	0.0827*	0.582 (13)
H13B	0.15966	0.41317	0.24058	0.0827*	0.582 (13)
H14	0.24201	0.13605	0.21557	0.0990*	0.582 (13)
H15A	0.03394	0.22858	0.32222	0.1475*	0.582 (13)
H15B	0.09975	0.07737	0.31874	0.1475*	0.582 (13)
H13C	0.14649	0.22473	0.13991	0.1287*	0.418 (13)
H13D	0.11891	0.38861	0.17638	0.1287*	0.418 (13)
H14'	0.28116	0.39504	0.34775	0.1282*	0.418 (13)
H15C	0.06577	0.11433	0.29497	0.1994*	0.418 (13)
H15D	0.17691	0.23038	0.40964	0.1994*	0.418 (13)
H18'	0.63089	1.27896	0.51160	0.1721*	0.497 (12)
H19D	0.77309	1.28521	0.67290	0.2087*	0.497 (12)
H19E	0.64631	1.11707	0.61376	0.2087*	0.497 (12)
H19F	0.84687	1.15273	0.62735	0.2087*	0.497 (12)
H20D	0.87958	1.38400	0.45788	0.2775*	0.497 (12)
H20E	0.91970	1.44933	0.57987	0.2775*	0.497 (12)
H20F	0.99250	1.31624	0.53410	0.2775*	0.497 (12)

### Prop-2-enyl 6-amino-5-cyano-4-(4-isopropylphenyl)-2-methyl-4H-pyran-3-carboxylate Atomic displacement parameters (Å^2^)

**Table:**

	*U*^11^	*U*^22^	*U*^33^	*U*^12^	*U*^13^	*U*^23^
O1	0.0466 (12)	0.0585 (12)	0.0825 (14)	0.0271 (9)	0.0283 (10)	0.0420 (11)
O2	0.0509 (14)	0.0689 (14)	0.122 (2)	0.0299 (12)	0.0314 (14)	0.0406 (14)
O3	0.0523 (13)	0.0759 (14)	0.0897 (16)	0.0181 (11)	0.0314 (12)	0.0460 (13)
N1	0.086 (2)	0.0742 (19)	0.113 (3)	0.0430 (16)	0.055 (2)	0.0587 (19)
N2	0.0538 (16)	0.0673 (17)	0.102 (2)	0.0328 (14)	0.0406 (16)	0.0476 (17)
C1	0.098 (3)	0.074 (2)	0.078 (3)	0.026 (2)	0.034 (2)	0.008 (2)
C2	0.131 (4)	0.066 (2)	0.096 (3)	0.052 (2)	0.050 (3)	0.025 (2)
C3	0.099 (3)	0.064 (2)	0.070 (2)	0.0466 (19)	0.031 (2)	0.0261 (17)
C4	0.0488 (16)	0.0518 (16)	0.0612 (18)	0.0195 (13)	0.0254 (14)	0.0234 (14)
C5	0.096 (3)	0.098 (3)	0.068 (2)	0.058 (2)	0.010 (2)	0.013 (2)
C6	0.108 (4)	0.122 (4)	0.066 (2)	0.053 (3)	0.011 (2)	0.011 (2)
C7	0.0425 (15)	0.0491 (15)	0.0570 (16)	0.0230 (12)	0.0187 (13)	0.0244 (13)
C8	0.0497 (16)	0.0466 (15)	0.0600 (17)	0.0221 (13)	0.0240 (14)	0.0249 (13)
C9	0.0495 (17)	0.0468 (15)	0.0629 (18)	0.0200 (13)	0.0247 (14)	0.0267 (13)
C10	0.0494 (17)	0.0496 (15)	0.0599 (17)	0.0194 (13)	0.0203 (14)	0.0265 (13)
C11	0.0417 (15)	0.0456 (14)	0.0542 (16)	0.0165 (12)	0.0166 (12)	0.0200 (12)
C12	0.0498 (17)	0.0473 (15)	0.0643 (18)	0.0178 (13)	0.0238 (14)	0.0189 (14)
C13	0.058 (7)	0.069 (6)	0.085 (8)	0.023 (5)	0.026 (6)	0.024 (6)
C14	0.069 (4)	0.082 (5)	0.105 (6)	0.019 (4)	0.036 (4)	0.039 (5)
C15	0.117 (9)	0.145 (10)	0.111 (9)	−0.003 (7)	0.044 (8)	0.081 (8)
C16	0.0576 (18)	0.0534 (17)	0.075 (2)	0.0289 (14)	0.0332 (16)	0.0292 (16)
C17	0.066 (2)	0.0564 (18)	0.100 (3)	0.0272 (16)	0.0299 (19)	0.0418 (18)
C18	0.181 (7)	0.111 (4)	0.106 (4)	0.047 (4)	0.032 (4)	−0.025 (3)
C19	0.172 (13)	0.198 (15)	0.145 (14)	0.103 (12)	−0.039 (11)	−0.046 (12)
C20	0.139 (11)	0.246 (16)	0.171 (13)	0.074 (11)	0.006 (9)	−0.140 (12)
C13'	0.064 (13)	0.139 (18)	0.138 (18)	−0.004 (11)	0.059 (12)	0.086 (14)
C14'	0.104 (7)	0.103 (8)	0.124 (9)	0.017 (6)	0.067 (7)	0.031 (7)
C15'	0.170 (19)	0.189 (17)	0.133 (16)	0.021 (14)	0.057 (14)	0.057 (15)
C19'	0.183 (12)	0.126 (9)	0.073 (7)	−0.005 (8)	0.060 (7)	−0.011 (6)
C20'	0.245 (17)	0.102 (8)	0.124 (11)	−0.063 (10)	0.071 (11)	−0.026 (8)

### Prop-2-enyl 6-amino-5-cyano-4-(4-isopropylphenyl)-2-methyl-4H-pyran-3-carboxylate Geometric parameters (Å, º)

**Table:**

O1—C9	1.357 (3)	C2—H2	0.9301
O1—C10	1.390 (4)	C3—H3	0.9307
O2—C12	1.195 (4)	C5—H5	0.9298
O3—C12	1.325 (4)	C6—H6	0.9300
O3—C13	1.463 (12)	C7—H7	0.9801
O3—C13'	1.46 (2)	C13—H13A	0.9703
N1—C16	1.150 (4)	C13—H13B	0.9697
N2—C9	1.339 (5)	C13'—H13D	0.9711
N2—H2A	0.87 (4)	C13'—H13C	0.9692
N2—H2B	0.98 (4)	C14—H14	0.9300
C1—C2	1.365 (7)	C14'—H14'	0.9310
C1—C18	1.516 (8)	C15—H15A	0.9267
C1—C6	1.360 (7)	C15—H15B	0.9337
C2—C3	1.379 (6)	C15'—H15D	0.9278
C3—C4	1.368 (5)	C15'—H15C	0.9343
C4—C5	1.367 (5)	C17—H17C	0.9597
C4—C7	1.521 (4)	C17—H17B	0.9604
C5—C6	1.388 (6)	C17—H17A	0.9601
C7—C11	1.508 (4)	C18—H18	0.9802
C7—C8	1.517 (5)	C18—H18'	0.9803
C8—C16	1.410 (4)	C19—H19A	0.9622
C8—C9	1.345 (5)	C19—H19B	0.9570
C10—C11	1.328 (4)	C19—H19C	0.9604
C10—C17	1.491 (5)	C19'—H19D	0.9600
C11—C12	1.476 (4)	C19'—H19E	0.9594
C13—C14	1.438 (16)	C19'—H19F	0.9612
C13'—C14'	1.45 (3)	C20—H20B	0.9596
C14—C15	1.221 (19)	C20—H20C	0.9627
C14'—C15'	1.20 (4)	C20—H20A	0.9592
C18—C20	1.459 (18)	C20'—H20D	0.9602
C18—C19'	1.414 (12)	C20'—H20E	0.9600
C18—C20'	1.56 (2)	C20'—H20F	0.9599
C18—C19	1.38 (2)		
			
O2···N2^i^	2.896 (4)	H3···H7	2.3519
O2···C4	3.329 (4)	H5···H19A^iii^	2.3221
O2···C9^ii^	3.252 (4)	H5···C10	2.9559
O3···C17	2.878 (5)	H5···C11	2.7234
O1···H19C^iii^	2.8182	H5···C19^iii^	3.0616
O2···H7	2.4173	H6···C19	2.8860
O2···H2A^i^	2.04 (4)	H6···H18	2.4564
O2···H13D	2.2364	H6···H19A	2.2978
O2···H13A	2.4933	H6···C19'	2.6878
O2···H13B	2.6178	H6···H19F	2.1965
O3···H17B	2.5660	H7···O2	2.4173
O3···H17C	2.8087	H7···H3	2.3519
N1···N2^iv^	3.034 (4)	H7···H17C^ii^	2.4031
N2···N1^iv^	3.034 (4)	H13A···O2	2.4933
N2···O2^v^	2.896 (4)	H13A···N2^ii^	2.8123
N1···H13C^ii^	2.8399	H13A···H2B^ii^	2.5026
N1···H2B^iv^	2.08 (4)	H13B···H15A	2.3825
N2···H13A^ii^	2.8123	H13B···O2	2.6178
C3···C16	3.555 (5)	H13C···C16^ii^	2.8379
C4···O2	3.329 (4)	H13C···N1^ii^	2.8399
C5···C10	3.536 (5)	H13D···O2	2.2364
C8···C12^ii^	3.556 (4)	H13D···H17A^i^	2.5051
C9···O2^ii^	3.252 (4)	H14···C2^ix^	3.0317
C9···C12^ii^	3.559 (4)	H14···H2^ix^	2.4213
C10···C5	3.536 (5)	H14'···C20^vi^	2.6993
C12···C9^ii^	3.559 (4)	H14'···H20B^vi^	2.3565
C12···C8^ii^	3.556 (4)	H14'···H20C^vi^	2.2354
C13'···C16^ii^	3.59 (2)	H15A···H13B	2.3825
C14'···C20^vi^	3.59 (2)	H15D···H20A^ix^	2.4522
C16···C13'^ii^	3.59 (2)	H17A···H13D^v^	2.5051
C16···C3	3.555 (5)	H17B···H2^ix^	2.4636
C17···O3	2.878 (5)	H17B···O3	2.5660
C20···C14'^vi^	3.59 (2)	H17B···C2^ix^	2.9158
C20'···C20'^vii^	3.04 (3)	H17C···H7^ii^	2.4031
C1···H19E^vi^	2.8777	H17C···O3	2.8087
C2···H20D	2.9839	H18···H6	2.4564
C2···H19E^vi^	2.8064	H18'···H2	2.3873
C2···H14^viii^	3.0317	H19A···H6	2.2978
C2···H20A	2.6244	H19A···C5^iii^	3.0573
C2···H17B^viii^	2.9158	H19A···H5^iii^	2.3221
C3···H19E^vi^	3.0721	H19A···C6	2.6516
C5···H19A^iii^	3.0573	H19B···H20C	2.5807
C6···H19A	2.6516	H19C···O1^iii^	2.8182
C6···H19E	3.0917	H19C···H20B	2.5807
C6···H20F	3.0572	H19D···H20E	2.4440
C6···H19F	2.7468	H19E···C6	3.0917
C10···H5	2.9559	H19E···C3^vi^	3.0721
C11···H5	2.7234	H19E···C1^vi^	2.8777
C14···H2^ix^	2.9748	H19E···C2^vi^	2.8064
C15'···H20A^ix^	2.9167	H19F···H20F	2.4391
C16···H13C^ii^	2.8379	H19F···C6	2.7468
C16···H2B	2.61 (4)	H19F···H6	2.1965
C19···H5^iii^	3.0616	H20A···C15'^viii^	2.9167
C19···H6	2.8860	H20A···H15D^viii^	2.4522
C19'···H6	2.6878	H20A···H2	2.2222
C20···H14'^vi^	2.6993	H20A···C2	2.6244
C20···H2	2.6308	H20B···H19C	2.5807
C20'···H20D^vii^	2.5639	H20B···H14'^vi^	2.3565
C20'···H20E^vii^	2.8570	H20C···H14'^vi^	2.2354
H2···C14^viii^	2.9748	H20C···H19B	2.5807
H2···C20	2.6308	H20D···C2	2.9839
H2···H14^viii^	2.4213	H20D···C20'^vii^	2.5639
H2···H17B^viii^	2.4636	H20D···H20D^vii^	2.3935
H2···H20A	2.2222	H20D···H20E^vii^	2.2045
H2···H18'	2.3873	H20E···H19D	2.4440
H2A···O2^v^	2.04 (4)	H20E···C20'^vii^	2.8570
H2B···H13A^ii^	2.5026	H20E···H20D^vii^	2.2045
H2B···C16	2.61 (4)	H20F···C6	3.0572
H2B···N1^iv^	2.08 (4)	H20F···H19F	2.4391
			
C9—O1—C10	119.4 (3)	O3—C13—H13A	109.46
C12—O3—C13	114.7 (6)	O3—C13—H13B	109.50
C12—O3—C13'	116.6 (10)	C14—C13—H13A	109.52
H2A—N2—H2B	121 (3)	C14—C13—H13B	109.58
C9—N2—H2A	119 (2)	H13A—C13—H13B	108.08
C9—N2—H2B	119 (2)	C14'—C13'—H13C	110.52
C2—C1—C6	116.3 (4)	C14'—C13'—H13D	110.51
C2—C1—C18	121.3 (5)	H13C—C13'—H13D	108.65
C6—C1—C18	122.4 (4)	O3—C13'—H13C	110.50
C1—C2—C3	122.1 (4)	O3—C13'—H13D	110.37
C2—C3—C4	121.1 (4)	C15—C14—H14	116.32
C3—C4—C5	117.6 (3)	C13—C14—H14	116.14
C3—C4—C7	120.6 (3)	C15'—C14'—H14'	110.37
C5—C4—C7	121.7 (3)	C13'—C14'—H14'	110.46
C4—C5—C6	120.3 (4)	H15A—C15—H15B	119.96
C1—C6—C5	122.6 (4)	C14—C15—H15B	119.71
C8—C7—C11	108.7 (2)	C14—C15—H15A	120.33
C4—C7—C8	110.9 (2)	C14'—C15'—H15D	120.44
C4—C7—C11	112.7 (2)	C14'—C15'—H15C	119.80
C7—C8—C9	121.5 (3)	H15C—C15'—H15D	119.76
C7—C8—C16	118.1 (3)	H17A—C17—H17B	109.43
C9—C8—C16	120.1 (3)	H17A—C17—H17C	109.49
O1—C9—N2	110.6 (3)	H17B—C17—H17C	109.46
O1—C9—C8	121.1 (3)	C10—C17—H17A	109.50
N2—C9—C8	128.2 (3)	C10—C17—H17B	109.46
O1—C10—C17	107.7 (3)	C10—C17—H17C	109.49
O1—C10—C11	121.3 (3)	C1—C18—H18	96.30
C11—C10—C17	131.0 (3)	C1—C18—H18'	108.26
C7—C11—C10	122.0 (3)	C19—C18—H18	96.37
C7—C11—C12	113.0 (2)	C20'—C18—H18'	108.22
C10—C11—C12	125.0 (3)	C20—C18—H18	96.16
O3—C12—C11	115.7 (3)	C19'—C18—H18'	108.15
O2—C12—C11	121.7 (3)	C18—C19—H19A	109.29
O2—C12—O3	122.6 (3)	H19A—C19—H19B	109.53
O3—C13—C14	110.6 (8)	C18—C19—H19B	109.64
O3—C13'—C14'	106.3 (14)	C18—C19—H19C	109.44
C13—C14—C15	127.5 (12)	H19B—C19—H19C	109.68
C13'—C14'—C15'	139 (2)	H19A—C19—H19C	109.25
N1—C16—C8	178.6 (4)	C18—C19'—H19E	109.55
C19—C18—C20	120.6 (11)	C18—C19'—H19D	109.54
C19'—C18—C20'	109.8 (10)	H19D—C19'—H19F	109.37
C1—C18—C19	119.1 (10)	C18—C19'—H19F	109.43
C1—C18—C20	116.8 (8)	H19D—C19'—H19E	109.52
C1—C18—C19'	116.9 (7)	H19E—C19'—H19F	109.42
C1—C18—C20'	105.3 (8)	C18—C20—H20B	109.63
C1—C2—H2	118.93	C18—C20—H20C	109.41
C3—C2—H2	119.01	C18—C20—H20A	109.62
C2—C3—H3	119.47	H20A—C20—H20C	109.31
C4—C3—H3	119.44	H20B—C20—H20C	109.28
C4—C5—H5	119.86	H20A—C20—H20B	109.58
C6—C5—H5	119.82	C18—C20'—H20D	109.46
C1—C6—H6	118.71	C18—C20'—H20E	109.50
C5—C6—H6	118.71	C18—C20'—H20F	109.48
C4—C7—H7	108.15	H20D—C20'—H20E	109.45
C8—C7—H7	108.09	H20D—C20'—H20F	109.46
C11—C7—H7	108.13	H20E—C20'—H20F	109.49
			
C10—O1—C9—C8	12.3 (4)	C5—C4—C7—C11	43.5 (5)
C9—O1—C10—C11	−17.4 (4)	C4—C5—C6—C1	1.0 (8)
C9—O1—C10—C17	163.3 (2)	C11—C7—C8—C9	−23.8 (3)
C10—O1—C9—N2	−168.6 (3)	C11—C7—C8—C16	161.7 (3)
C12—O3—C13—C14	174.0 (7)	C4—C7—C8—C16	−73.9 (3)
C13—O3—C12—O2	1.6 (7)	C4—C7—C8—C9	100.7 (3)
C13—O3—C12—C11	179.9 (6)	C4—C7—C11—C12	76.1 (3)
C2—C1—C18—C19	−129.6 (10)	C8—C7—C11—C10	19.0 (3)
C6—C1—C2—C3	−0.7 (7)	C4—C7—C11—C10	−104.4 (3)
C18—C1—C6—C5	−178.0 (5)	C8—C7—C11—C12	−160.5 (2)
C18—C1—C2—C3	177.7 (5)	C7—C8—C9—N2	−169.3 (3)
C6—C1—C18—C20	−152.4 (9)	C16—C8—C9—O1	−176.0 (3)
C2—C1—C6—C5	0.4 (8)	C16—C8—C9—N2	5.1 (5)
C2—C1—C18—C20	29.3 (11)	C7—C8—C9—O1	9.6 (4)
C6—C1—C18—C19	48.7 (12)	O1—C10—C11—C12	179.3 (2)
C1—C2—C3—C4	−0.3 (7)	C17—C10—C11—C7	179.1 (3)
C2—C3—C4—C5	1.7 (6)	C17—C10—C11—C12	−1.5 (5)
C2—C3—C4—C7	−176.0 (4)	O1—C10—C11—C7	−0.1 (4)
C3—C4—C7—C11	−138.9 (3)	C10—C11—C12—O3	23.7 (4)
C3—C4—C5—C6	−2.0 (6)	C7—C11—C12—O2	21.4 (4)
C3—C4—C7—C8	98.9 (4)	C10—C11—C12—O2	−158.0 (3)
C7—C4—C5—C6	175.7 (4)	C7—C11—C12—O3	−156.9 (2)
C5—C4—C7—C8	−78.7 (4)	O3—C13—C14—C15	139.9 (13)

Symmetry codes: (i) *x*−1, *y*, *z*; (ii) −*x*+1, −*y*+1, −*z*; (iii) −*x*+2, −*y*+2, −*z*+1; (iv) −*x*+2, −*y*+2, −*z*; (v) *x*+1, *y*, *z*; (vi) −*x*+1, −*y*+2, −*z*+1; (vii) −*x*+2, −*y*+3, −*z*+1; (viii) *x*, *y*+1, *z*; (ix) *x*, *y*−1, *z*.

### Prop-2-enyl 6-amino-5-cyano-4-(4-isopropylphenyl)-2-methyl-4H-pyran-3-carboxylate Hydrogen-bond geometry (Å, º)

**Table:**

*D*—H···*A*	*D*—H	H···*A*	*D*···*A*	*D*—H···*A*
N2—H2*A*···O2^v^	0.87 (4)	2.04 (4)	2.896 (4)	170 (3)
N2—H2*B*···N1^iv^	0.98 (4)	2.08 (4)	3.034 (4)	163 (3)

Symmetry codes: (iv) −*x*+2, −*y*+2, −*z*; (v) *x*+1, *y*, *z*.
